# Fabrication and characterization of spin-coated multilayer surface-eroding implants for automated multi-pulse drug delivery

**DOI:** 10.1007/s10544-026-00848-4

**Published:** 2026-09-03

**Authors:** Parker R. Brewster, Katherine Nevils, Lilly Ates, Acelynn Sellers, Heath Stevens, Omayma Alazzam, Glenn M. Walker, Thomas A. Werfel

**Affiliations:** 1 Department of Biomedical Engineering, University of Mississippi, University, MS 38677 USA; 2 Department of Chemical Engineering, University of Mississippi, University, MS 38677 USA; 3 Department of Mechanical Engineering, University of Mississippi, University, MS 38677 USA; 4 Department of BioMolecular Sciences, University of Mississippi, University, MS 38677 USA

**Keywords:** Pulsatile drug delivery, Spin coating, Surface-eroding polymers, Multilayer implant, Cellulose acetate phthalate

## Abstract

Medication nonadherence contributes to disease progression, avoidable hospitalization, and an estimated $100–300 billion in annual excess healthcare costs in the United States. Implants that encode a dosing schedule during fabrication offer an alternative to patient-dependent administration. Here we apply spin coating to build multilayer surface-eroding implants from cellulose acetate phthalate (CAP) and Pluronic F-127, stacking fluorescein-loaded poly(vinyl alcohol) active layers between degradable CAP–Pluronic composite (CAPP) barrier layers whose thickness sets the interval between release events. Spin-curve calibration gave an inverse power-law dependence of thickness on rotational speed (R² = 0.979) from 436 ± 9 to 92 ± 4 μm. Against solvent-cast films produced from a necessarily different formulation at a matched 400 μm nominal target, spin coating reduced batch-to-batch standard deviation from approximately 34 to 2 μm and within-film standard deviation from 43–47 to 5–12 μm (*n* = 3 independently fabricated films per method); at a 100 μm target it reduced the maximum surface excursion below the mean plane, measured by atomic force microscopy, from 146.1 to 4.5 nm; confocal Raman mapping showed the standard deviation of the CAP/F-127 peak-height ratio falling from 2.91 to 0.77. Two device configurations, designated Q16 and Q72 after the approximately 16 and 72 h inter-pulse intervals they produced, each gave three discrete release events with near-baseline inter-pulse signal (*n* = 6 devices per condition).

**Graphical abstract:**

**Figure:**
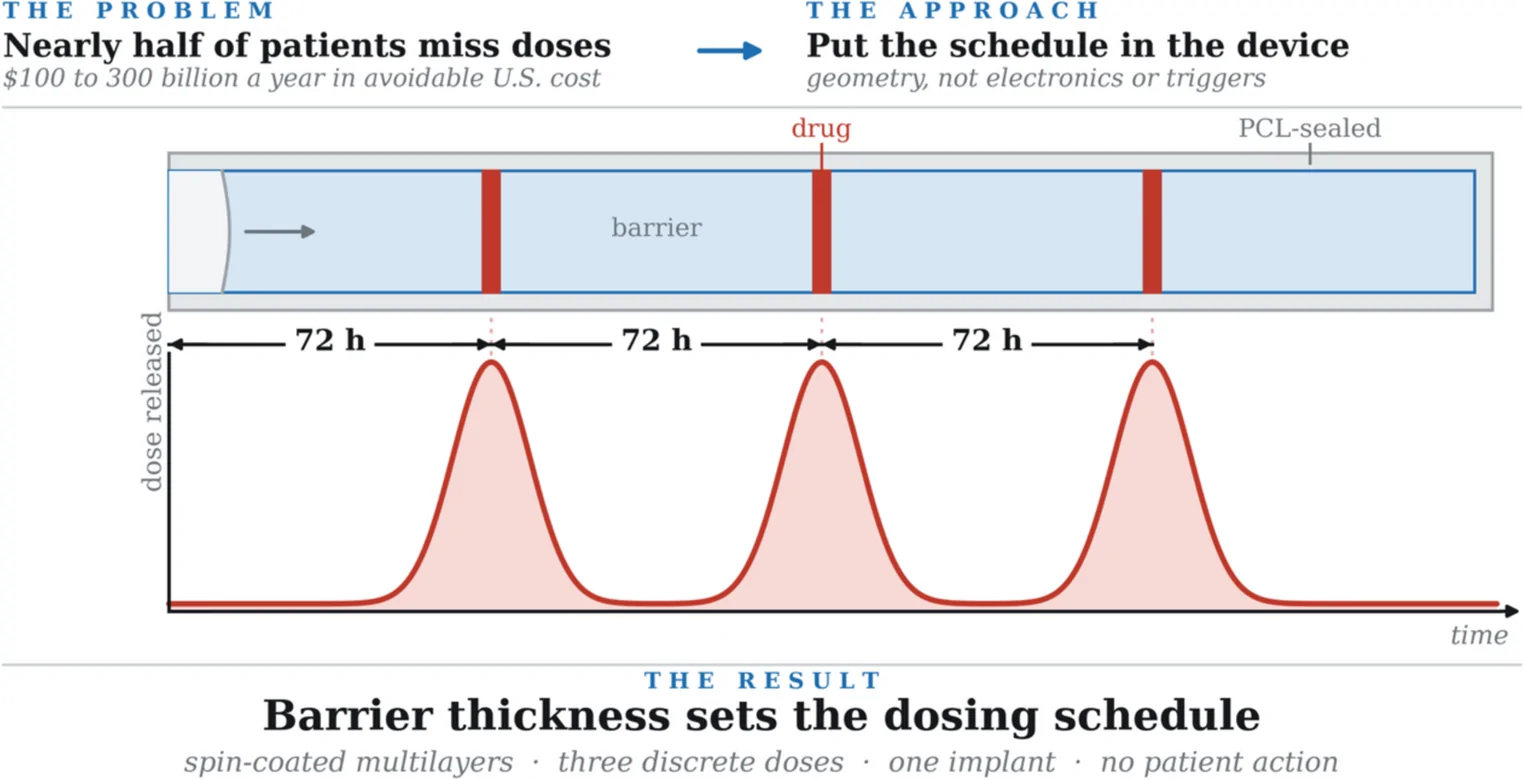
A dosing schedule encoded in device geometry rather than in patient behavior. CAP–Pluronic F-127 (CAPP) barrier layers and fluorescein-loaded poly(vinyl alcohol) active layers are stacked by sequential spin coating; the completed stack is encapsulated in polycaprolactone on every face except one. Erosion advances inward from that face alone, releasing a dose each time the front reaches an active layer, so the thickness of each barrier layer sets the interval to the next release event. Devices built to a nominal 72 h inter-pulse design target released three discrete, temporally separated pulses at 72, 144 and 216 h in vitro (n = 6 devices), the first of these set by a blank unit at the exposed face. The cross-section is schematic, layer thicknesses are not drawn to scale, and the release trace is illustrative.

## Introduction

Medication nonadherence remains one of the most persistent barriers to effective therapy, contributing to treatment failure, hospitalization, and avoidable mortality in chronic disease management. Beyond its clinical toll, medication nonadherence is estimated to account for $100–300 billion annually in avoidable U.S. healthcare costs (Cutler et al. 2018). Approximately 50% of patients with chronic illnesses do not take medications as prescribed, a statistic that has remained consistent across decades of analysis (Osterberg and Blaschke 2005; World Health Organization 2003). Among U.S. adults aged 40–79, 69.0% take at least one prescription medication, and 22.4% take five or more (Hales et al. 2019). Nonadherence has been associated with increased hospitalization risk and higher mortality in conditions including cardiovascular disease, diabetes, and psychiatric disorders (Ho et al. 2006; Osterberg and Blaschke 2005). The resulting gaps in dosing produce fluctuating plasma drug concentrations, reduced therapeutic efficacy, and preventable adverse effects. For conditions requiring precise temporal exposure, such as psychiatric treatment, endocrine therapies, and antimicrobial regimens, missed doses can significantly compromise clinical outcomes (Vrijens et al. 2012).

Many physiological processes exhibit strong temporal regulation, and continuous drug exposure is not always pharmacologically optimal. Endogenous signaling molecules such as insulin, cortisol, growth hormone, and gonadotropin-releasing hormone are secreted in pulsatile patterns that regulate receptor signaling and downstream biological responses (Veldhuis 2008). Many diseases similarly display circadian variation in symptom severity, with asthma, early-morning blood pressure surges, rheumatoid arthritis, and cardiovascular events following time-dependent patterns linked to endogenous rhythms (Smolensky and Peppas 2007). Aligning drug administration with these rhythms can improve therapeutic outcomes and reduce toxicity in chronotherapeutic contexts (Levi et al. 2010). Beyond chronotherapy, the temporal pattern of exposure can itself shape pharmacologic response: continuous receptor activation drives desensitization and downstream adaptations that diminish efficacy over time (Allouche et al. 2014). Clinical observations similarly suggest that intermittent bolus administration may reduce dose escalation relative to continuous infusion (Gourlay et al. 1991), and tolerance that develops during continuous infusion can even be reversed by transitioning to pulsatile delivery (Heetla et al. 2009). Pulsatile drug delivery systems have therefore emerged to generate discrete, time-controlled bursts of release following programmable lag periods (Bussemer et al. 2001; Smolensky and Peppas 2007). These systems embed dosing schedules directly within device architecture to automate therapeutic exposure while reducing reliance on patient adherence, and have been explored for chronotherapeutic treatment of asthma, hypertension, arthritis, and psychiatric disorders, where symptom severity follows circadian variation (Roy and Shahiwala 2009). However, many reported pulsatile systems rely on diffusion-controlled matrices, swelling-driven mechanisms, osmotic rupture, or pH-responsive coatings that are sensitive to environmental variability and often present challenges in achieving reproducible multi-pulse timing across extended dosing schedules (Arora et al. 2006).

Across the pulsatile drug delivery literature, several distinct architectural strategies have been explored to generate discrete release events (Brewster et al. 2023). These span a wide range of scales, from molecularly engineered soft polymeric nanocarriers, whose design is increasingly guided by computational and machine learning methods (Basak 2026b), to implantable devices that carry a fixed dosing schedule. Early implantable reservoir systems pioneered by the Langer and Cima groups demonstrated that microfabricated devices containing sealed drug reservoirs could be opened in a programmed manner, establishing the feasibility of embedding multi-dose regimens within an implant rather than relying on patient adherence (Santini et al. 1999). Santini et al. first reported a solid-state microchip capable of controlled release from individual reservoirs, and subsequent biodegradable and multireservoir iterations achieved multiple sequential pulses in vitro and in vivo, with later work extending these platforms to chronic programmed peptide delivery in animal models and ultimately to first-in-human testing of a wirelessly controlled implant capable of releasing discrete daily doses on a predefined schedule (Farra et al. 2012; Prescott et al. 2006; Richards Grayson et al. 2003). In parallel, oral pulsatile systems such as time-controlled explosion systems, osmotic rupture capsules, and compression-coated tablets were developed to generate lag times followed by rapid drug release, often for chronotherapeutic applications (Hata et al. 1994).

While these approaches demonstrated the conceptual feasibility of pulsatile dosing, both implantable and oral platforms can face practical constraints on the achievable number of discrete pulses per device. Microreservoir systems are often limited by the volume required to house individual sealed compartments and associated membranes or electronics, and rupture-based oral systems are typically designed for one or two burst events before matrix depletion. Multilayer surface-eroding architectures offer a complementary approach, defining each dosing event through the thickness of sequential barrier layers rather than through individually sealed reservoirs: drug-containing layers are separated by degradable barriers, and release proceeds as the surface erodes through the stack in a predetermined sequence. Because dosing events are encoded within the vertical layer structure, pulse number can in principle be set by the number of stacked layers rather than by discrete reservoir compartments, reducing reliance on individual housing structures, membranes, or electronic triggering elements, although extending this approach to larger numbers of pulses remains to be demonstrated.

Cellulose acetate phthalate (CAP) is one such surface-eroding polymer platform well suited to pulsed release. With a long history of pharmaceutical use, CAP forms compatible composites with Pluronic F-127 whose erosion can shift from diffusion-dominated release under acidic conditions to surface-erosion-controlled release under physiological pH, and early multilaminate constructs with alternating drug-loaded layers showed that pulsatile release could be generated through sequential erosion of the stack (Xu and Lee 1993). Studies by Puleo and colleagues further characterized CAP-based degradable composites and related cellulose-derived polymers, relating polymer composition, hydrophilicity, mechanical properties, and degradation behavior, and establishing them as versatile surface-eroding delivery materials (Jeon et al. 2007; Raiche and Puleo 2003; Sundararaj et al. 2013). Building on this foundation, our recent work showed that multilayer CAPP films can encode discrete dosing schedules within implantable devices, achieving interval delivery of 5HT2A agonists and establishing multilayer CAPP as a programmable platform for interval drug administration (Hossain et al. 2023).

These early multilayer CAPP devices were fabricated by solvent casting individual polymer films followed by manual stacking to create alternating barrier and active layers. While this approach demonstrated the feasibility of multilayer surface-eroding pulsatile systems, solvent casting can produce variability in film thickness and may introduce partial interlayer mixing during assembly. These effects complicate precise control over barrier thickness, surface roughness, and compositional homogeneity of the polymer blend, all of which directly govern the programmed lag times that define sequential drug release events, motivating fabrication methods capable of producing uniform films with well-defined interfaces.

In the architecture described here, thin poly(vinyl alcohol) (PVA) films loaded with a fluorescent tracer serve as the drug-containing active layers, interleaved between thicker CAPP barrier layers whose thickness defines the erosion interval preceding each release event. Spin coating offers a tunable thin-film deposition method in which centrifugal force and solvent evaporation determine final film thickness as a function of solution viscosity, rotational speed, and concentration (Meyerhofer 1978; Routh and Russel 1998), a relationship that has been verified empirically for free-standing biodegradable polymer films (Allaf 2025), enabling sequential deposition of alternating active and blank layers with controlled thickness and minimal mechanical disturbance to previously deposited films. In this study, we applied spin coating to fabricate multilayer CAPP constructs intended for pulsatile drug release, seeking to determine how fabrication parameters influence film morphology, interlayer integrity, and thickness reproducibility, and how layer thickness governs erosion-mediated drug release timing in stacked architectures. To assess architectural precision, multilayer constructs containing multiple active layers were fabricated and characterized structurally and functionally, establishing a framework for engineering multilayer, surface-eroding implants in which pulse timing and dosing interval are controlled by the spin coating process.

Taken together, the literature identifies a specific and unresolved gap. Reservoir-based implants achieve programmable multi-pulse dosing but bound the achievable pulse count by the volume and sealing hardware required per compartment and typically depend on electronics or an actively triggered membrane (Farra et al. 2012; Prescott et al. 2006; Richards Grayson et al. 2003; Saha et al. 2025; Santini et al. 1999; Ullah et al. 2025). Oral rupture- and coating-based systems avoid that hardware but are generally limited to one or two burst events and remain sensitive to gastrointestinal pH, transit, and hydration variability (Arora et al. 2006; Bussemer et al. 2001; Hata et al. 1994). Multilayer surface-eroding stacks resolve both constraints in principle, because pulse count is set by layer count and timing is set by barrier thickness, but the fabrication route reported for such devices to date has been solvent casting of individual films followed by manual assembly, which delivers neither the thickness precision nor the interfacial definition that geometric timing control requires (Hossain et al. 2023; Xu and Lee 1993). Related film- and mold-based routes to implantable and transdermal polymer delivery devices share this constraint, relying on casting or molding rather than on a deposition step whose thickness is set directly by process parameters (Castro et al. 2025; Mao et al. 2024). The unmet need is therefore not a new release mechanism but a deposition method able to place barrier and active layers at specified thickness with defined interfaces, repeatably, in a single sequential process.

We are not aware of a previous application of spin coating to the sequential fabrication of multilayer surface-eroding constructs for pulsatile drug delivery; reported use of spin coating in drug delivery has largely concerned single-layer films and surface coatings rather than stacked architectures in which each layer thickness encodes a discrete dosing event (Allaf 2025; He et al. 2024). The specific contributions of the present work are therefore fourfold: (i) a calibrated spin-curve design space for the CAP–Pluronic F-127 system spanning approximately 15 to 436 μm, established across three solution formulations; (ii) a quantitative, multi-modal comparison of spin coating against solvent evaporation casting at a matched nominal thickness, using thickness metrology, atomic force microscopy, and confocal Raman mapping rather than a single descriptor, with the limitation that the two routes necessarily differ in solvent, concentration and drying conditions, so the comparison is between two complete fabrication routes rather than between deposition mechanisms alone; (iii) demonstration that a solvent-orthogonal deposition sequence permits aqueous PVA active layers to be spun directly onto gVL-based CAPP barrier layers with no interlayer mixing detectable at the magnifications imaged, which is what makes sequential in situ stacking possible at all; and (iv) functional demonstration that inter-pulse timing is set by fabrication-defined barrier thickness across two barrier thicknesses differing six-fold, together with an account of where that mapping breaks down.

## Materials and methods

### Materials

Cellulose acetate phthalate (CAP; Supelco, Cat. 22192-500G-F), Pluronic F-127 (Sigma-Aldrich, Cat. P2443-250G), phosphate-buffered saline (PBS tablets, pH 7.4; EMD Millipore, Cat. 524650-1EA), acetone (Fisher Chemical, Cat. A18-4, ACS grade), γ-valerolactone (gVL; Sigma-Aldrich, Cat. 918660–1 L, ≥ 99%), poly(vinyl alcohol) (PVA; Sigma-Aldrich, Cat. 360627-500G, M_w_ 9,000–10,000, 80% hydrolyzed), polycaprolactone (PCL; Sigma-Aldrich, Cat. 440744, M_n_ 80,000), chloroform (Alfa Aesar, Cat. C298-4 L, ACS grade, ethanol-stabilized), fluorescein disodium salt (Acros Organics, Cat. 17324 − 1000), and rhodamine B (Sigma-Aldrich, Cat. R6626) were used as received. Deionized water was produced in-house using a Milli-Q IQ 7000 ultrapure water system (MilliporeSigma). Silicon wafers (UniversityWafer, South Boston, MA, USA; Item 1080, 76.2 mm diameter, 400 μm thick, single-side polished, mechanical grade) were used as deposition substrates. Fluorescence measurements were performed using an Agilent Synergy H1 microplate reader.

### Spin-curve characterization

To establish the relationship between rotational speed and film thickness for the CAPP system, spin curves were generated by systematically varying rotational speed while holding spin time constant at 30 s. A 90:10 (w/w) CAP: Pluronic F-127 formulation dissolved at 60% (w/v) in gVL was used as the primary fabrication formulation; additional curves were generated for CAPP dissolved in gVL at 40% (w/v) and for CAPP dissolved in a 1:1 gVL: acetone mixture at 10% (w/v) to characterize the effect of solution concentration and solvent composition on final film thickness. Rotational speed was varied across the operational range of the spin coater, from the minimum speed required to produce continuous films to speeds beyond which further increases in rpm produced negligible changes in thickness; rpm increments ranged from 25 to 250 rpm depending on the region of the curve being characterized. For each speed, film thickness was measured at five locations on a single film using a digital dial indicator and averaged to generate each point on the spin curve. These five values are repeated measurements taken within one film rather than measurements of five independently fabricated films, so the associated standard deviation describes within-film measurement dispersion and not film-to-film reproducibility (see Section 2.12). A power-law regression was applied to the varying region of the primary 60% gVL curve to quantify the thickness-speed relationship and establish calibration values for subsequent fabrication.

### Fabrication of CAPP films: spin coating

CAP and Pluronic F-127 were combined at a 90:10 (w/w) ratio and dissolved in gVL at 60% (w/v). The ratio was selected on the basis of prior characterization of CAP–Pluronic composites, in which Pluronic F-127 acts as a hydrophilic pore-forming and plasticizing additive whose weight fraction governs water uptake, erosion rate, and film mechanical properties, and in which low Pluronic fractions preserve surface-erosion-dominated behavior at physiological pH while improving film handling and reducing brittleness (Jeon et al. 2007; Raiche and Puleo 2003; Sundararaj et al. 2013; Xu and Lee 1993). The same composition was used in our previous multilayer CAPP devices (Hossain et al. 2023) and was retained here deliberately, so that the fabrication comparison would be performed at a formulation whose erosion behavior had already been characterized rather than confounded with a simultaneous change in formulation. The mixture was sealed and held at 50 °C for 24 h until fully dissolved. For single-layer release studies, rhodamine B was added at 1% (w/v solvent) prior to deposition. A 10 mL aliquot of polymer solution was deposited onto a silicon wafer and spun at 1050 rpm for 30 s to produce approximately 200 μm films, or at 1750 rpm for 30 s to produce approximately 100 μm films. Two sequential 200 μm films were used to create 400 μm films. Following deposition, wafers were placed on a hot plate at 90 °C for 2 h to ensure complete solvent evaporation. Dried films were gently removed from the wafer using tweezers and punched into circular samples using a single-hole punch. Film thickness was verified with a digital dial indicator.

### Fabrication of CAPP films: solvent evaporation casting

CAP and Pluronic F-127 (90:10 w/w) were dissolved in acetone at 7% (w/v) with periodic vortexing for 4 h to yield a homogeneous solution. Rhodamine B was added at 1% (w/v solvent) prior to casting. To produce 400 μm films, 26 mL of polymer solution was poured into a 4-inch diameter glass Petri dish; 6 mL produced 100 μm films under identical conditions. Dishes were left uncovered at room temperature (21 to 23 °C) for 24 h to allow solvent evaporation. After drying, films were removed from the Petri dish using tweezers, punched into circular samples, and measured for thickness with a digital dial indicator. The solvent-cast formulation differs from the spin-coated formulation in four respects: solvent (acetone versus gVL), polymer concentration (7% versus 60% w/v), drying temperature (21–23 °C versus 90 °C), and drying duration (24 h versus 2 h). These parameters were not matched, because each deposition method requires a solution viscosity and evaporation rate compatible with its own physics: solvent casting requires a dilute, volatile solution that levels under gravity and dries without skinning, whereas spin coating requires a concentrated, higher-viscosity solution in a low-volatility solvent that does not evaporate before centrifugal spreading is complete. Each formulation therefore represents the condition under which its respective method yields usable films at the target thicknesses studied here, and the comparison that follows is a comparison of two complete fabrication routes as they would realistically be implemented, not a controlled isolation of the deposition step.

### Fabrication of multilayered devices: spin coating

**Fig. 1:**
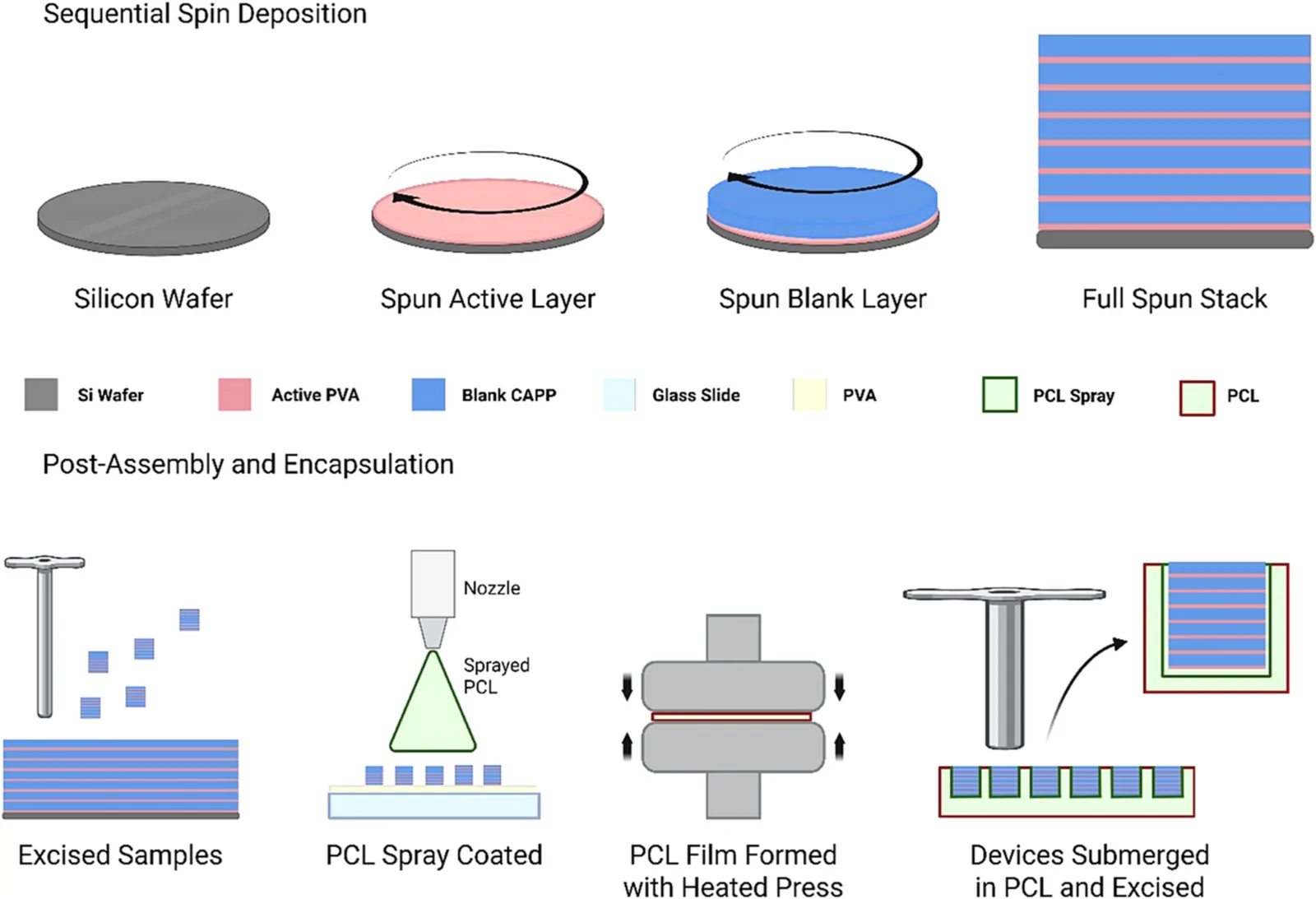
Schematic overview of multilayered pulsatile device fabrication. (Top) sequential spin deposition of alternating blank CAPP (blue) and fluorescein-loaded PVA active (pink) layers onto a silicon wafer substrate, repeated to yield a multilayered stack of the desired architecture, shown as a cross-sectional schematic. (Bottom, left to right) completed layer stacks are adhered to a glass slide using PVA adhesive; devices are spray-coated with a dilute PCL solution (1% w/v in chloroform: acetone, 1:1 v/v) deposited via nozzle to form a thin encapsulating film; the coated devices are subjected to heated compression pressing within a bulk PCL matrix; individual devices are excised using a biopsy punch, yielding final constructs encased within a PCL shell on all faces except the defined erosion surface

Multilayered constructs were produced by sequentially spinning alternating blank CAPP layers and fluorescein-loaded PVA active layers onto a silicon wafer (Fig. 1). Blank layers were fabricated from unloaded 90:10 (w/w) CAP: Pluronic F-127 dissolved at 60% (w/v) in gVL. Each inter-pulse blank unit for the devices designated Q16, after the approximately 16 h inter-pulse interval they produced, consisted of two sequential depositions at 1050 rpm for 30 s, each followed by drying at 90 °C for 2 h, yielding a nominal combined blank thickness of 400 μm per inter-pulse unit. For the devices designated Q72, after the 72 h inter-pulse interval they produced, twelve sequential 200 μm depositions were used per inter-pulse blank unit, yielding a nominal combined blank thickness of 2.4 mm. The two conditions differ in the layer that forms the exposed erosion face. In Q72 devices a full blank unit occupies that position, so a 2.4 mm blank layer must erode before the first active layer is reached; each Q72 device therefore comprises four blank units and three active layers, a total of 48 blank depositions. In Q16 devices an active layer occupies that position, so the drug-loaded layer is exposed at the outset; each Q16 device comprises three 400 μm blank units and three active layers. In both conditions the deepest blank unit serves as a backing layer and takes no part in release timing. Solvent removal prior to release testing differed between the two device conditions reported here. Q72 devices were vacuum dried at room temperature for 24 h between encapsulation and the start of release testing. Q16 devices were held at room temperature overnight to dry before testing. Residual solvent in an assembled device is expected to be a blend of indeterminate composition: gVL from the CAPP depositions predominates, but the chloroform: acetone mixture used for the PCL spray coat can penetrate the bulk during coating. Because removal of these solvents from the bulk is slow and depends on humidity and on their respective vapor pressures rather than on elapsed time, an extended reduced-pressure step at room temperature was subsequently adopted.

Active layers were prepared by dissolving PVA in deionized water at 40% (w/v) with fluorescein added at 1% w/v prior to deposition. A 5 mL aliquot of active layer solution was deposited onto the dried blank surface and spun at 2000 rpm for 30 s to produce active layers of approximately 10 μm. Active layers were dried at room temperature for 12 h before the next blank deposition cycle was initiated. This alternating blank-active deposition sequence was repeated to achieve the desired number of active layers per device.

Following stack assembly, completed devices were adhered to glass slides using 40% (w/v) PVA as an adhesive layer. Devices were then spray-coated with a 1% (w/v) PCL solution dissolved in a 1:1 (v/v) chloroform: acetone mixture to deposit a thin encapsulating film (less than 10 μm) that served as a bonding layer, followed by overnight drying under vacuum. Devices were subsequently embedded within 10 mm thick PCL films formed by heated compression pressing, providing a thicker, more mechanically stable outer shell that adhered to the spray-coated bonding layer. Individual devices were excised from the PCL film with a surrounding border of PCL retained on all encapsulated faces, such that each device remained encased within a protective PCL shell on all sides except the single exposed PVA adhesive face, which served as the defined erosion surface through which directional degradation proceeds sequentially through the layer stack. The interval between immersion and the first release event is set entirely by whether a blank unit occupies that face.

### Thickness and dimensional characterization

Film thickness uniformity was evaluated using three independently fabricated spin-coated films and three solvent-cast films, each at a target thickness of 400 μm. Twenty samples (6 mm in diameter) were excised from each film and measured individually using a digital dial indicator, yielding 20 measurements per film. These 20 values are repeated measurements taken from within a single film rather than 20 independent samples; the independent fabrication replicate is the film (*n* = 3 films per method), and the 20 within-film measurements are treated as technical replicates nested within each film, giving 60 total measurements per method (see Section 2.12).

### Surface roughness: atomic force microscopy

Surface roughness of spin-coated and solvent-cast CAPP films was characterized by atomic force microscopy (AFM). Films were fabricated as described above at a target thickness of 100 μm for both methods. Circular specimens approximately 6 mm in diameter were excised from each film and mounted on aluminum sample discs for analysis. AFM imaging was performed using a Dimension FastScan system (Bruker, Billerica, MA, USA) operating in ScanAsyst mode with a FastScan-C cantilever probe. Topography images were acquired over scan areas of 1 × 1 μm, 5 × 5 μm, and 20 × 20 μm. Three non-overlapping regions were analyzed per sample to account for spatial variation across the film surface. Height data were processed using a third-order polynomial flattening procedure prior to analysis. Surface topography and the maximum excursion below the mean plane were compared between the two fabrication methods.

### Polymer homogeneity: Raman spectroscopy

Compositional homogeneity of spun and cast CAPP films was assessed by confocal Raman spectroscopy (HORIBA XploRA Plus, HORIBA Scientific, Edison, NJ) on film surfaces to characterize polymer distribution across both fabrication methods. Measurements were performed using a 532 nm excitation laser with vertical polarization, a 50× long-working-distance dark-field objective, a 1200 lines/mm grating, and confocal apertures of 100 μm (slit) and 300 μm (pinhole). Spectra were acquired over a range of 200 to 3199 cm⁻¹ at a spectral resolution of 15.7 cm⁻¹, with an exposure time of 4 s and four accumulations per point using a spike filter to reject cosmic ray artifacts; the detector was thermoelectrically cooled to -60 °C. Each film was mapped across a 0.3 × 0.3 mm area subdivided into a 3 × 3 grid of nine equally spaced measurement points. Homogeneity was assessed by calculating, at each point, the peak height of the CAP carbonyl stretch (1700 to 1800 cm⁻¹) divided by the peak height of the F-127 C-O-C stretch (840 to 880 cm⁻¹), each measured above a local linear baseline drawn between the endpoints of the respective integration window, with spatial uniformity of the resulting ratio across all nine points used as the index of compositional homogeneity.

One film per fabrication method was mapped, and the nine measurement points within a map are technical replicates nested within that film, so this Raman comparison rests on a single fabrication replicate per method (see Section 2.12).

### Layer interface visualization: scanning electron microscopy

Interfacial definition and individual layer dimensions were visualized by scanning electron microscopy (SEM). For each fabrication method, a three-layer construct with two 400 μm blank CAPP outer layers was fabricated to assess interfacial definition and layer morphology. For spin-coated samples, a 10 μm fluorescein-PVA active layer was spun between the two blank CAPP layers (400–10–400 μm) to isolate the active–blank interface. For solvent-cast samples, a 100 μm rhodamine-B-loaded CAPP film served as the middle layer between two 400 μm blank CAPP films (400–100–400 μm); the three films were cast separately by solvent evaporation and joined by acetone-mediated bonding, as solvent casting cannot reliably produce active layers as thin as those deposited by spin coating. This difference has three consequences. First, the two three-layer constructs are not matched in middle-layer composition or thickness: the spin-coated construct contains a 10 μm fluorescein-loaded PVA middle layer, whereas the solvent-cast construct contains a 100 μm rhodamine-B-loaded CAPP middle layer. The resulting images therefore illustrate the interfacial quality each fabrication route achieves at its own practical limit, and are not a one-to-one structural comparison of an otherwise identical construct. Second, the cast construct was assembled by bonding three separately cast films with acetone, whereas the spin-coated construct was built by sequential in situ deposition, so the assembly route differs in addition to the film-formation method. Third, the three-layer construct is the repeating unit from which the multilayer devices are assembled rather than a simplified analogue of them: a 400 μm blank unit is built from two sequential 200 μm depositions, and the construct therefore contains both of the interface types present in a complete device, the blank-on-blank interface between successive depositions and the blank-on-active interface. The complete devices present the same two interface types throughout: a Q16 device alternates three 400 μm blank units with three active layers, and a Q72 device alternates four blank units of twelve depositions each with three active layers. The constructs contain fewer layers than a complete device, so cumulative effects that appear only in deeper stacks would not be captured here. Samples were embedded in optimal cutting temperature (OCT) compound and sectioned using a cryotome to obtain cross-sections approximately 10 μm thick. Sections were mounted on aluminum stubs using double-sided conductive carbon tape and sputter-coated with a 10 nm gold-palladium (60:40) layer using a Desk V HP sputter coater (Denton Vacuum, Moorestown, NJ, USA) under argon atmosphere. Imaging was performed using a JSM-7200FLV field-emission scanning electron microscope (JEOL Ltd., Tokyo, Japan) at an accelerating voltage of 5 kV, working distances of 5 to 9 mm, and using a lower secondary electron detector. Images were collected at multiple magnifications to visualize overall layer structure, interfacial features, and surface morphology. Active layer thickness was measured at multiple locations along the interface for both fabrication methods to evaluate spatial variability.

### Drug release: single-layer films

Rhodamine B release from single-layer CAPP films was measured in PBS (pH 7.4) at 37 °C under constant agitation (vial shaker, 90 rpm). Two independently fabricated films per method and thickness were used, with three punched samples taken from each film for testing, yielding six specimens per condition across four conditions (spin-coated 400 μm, cast 400 μm, spin-coated 100 μm, and cast 100 μm). The three specimens taken from a given film are technical replicates nested within that film and are not independent fabrication replicates; the number of independent fabrication units per condition is therefore two, not six. To avoid pseudoreplication, these six specimens are treated throughout as repeated measurements drawn from two fabrication units, and the dispersion reported for them is descriptive rather than a basis for inference (see Section 2.12). Each sample was submerged in 18 mL PBS in an individual vial. Supernatant aliquots were collected every 4 h for 400 μm films and every 1 h for 100 μm films, with fresh PBS added to maintain sink conditions after each collection. Fluorescence intensity was measured at λ_ex_ = 540 nm, λ_em_ = 585 nm using an Agilent Synergy H1 microplate reader. Collection continued until fluorescence signal was no longer detectable above background, indicating complete film erosion.

### Drug release: multilayered devices

Pulsatile fluorescein release from multilayered devices was measured under the same PBS/37°C/90 rpm conditions described above. Six independently fabricated devices were tested per dosing interval condition (Q16 and Q72; *n* = 6 per condition). For Q16 devices, supernatant was collected at 4 h intervals over the full anticipated release duration; for Q72 devices, supernatant was collected at 12 h intervals. Fresh PBS was added after each collection to maintain sink conditions. Fluorescence intensity was measured at λ_ex_ = 490 nm, λ_em_ = 515 nm using an Agilent Synergy H1 microplate reader. Data were expressed as fluorescence intensity (RFU) over time and plotted for each device individually and as group means with standard deviation.

### Statistical analysis and definition of replicates

Throughout this study the independently fabricated film or device is treated as the independent experimental unit, and measurements taken from within a single film or device are treated as technical, repeated measurements nested within that unit. Specifically: (i) in the spin-curve characterization (Section 2.2), the five thickness values obtained at each rotational speed are repeated measurements at different locations on a single film, so the reported standard deviation describes within-film measurement dispersion; (ii) in the thickness uniformity comparison (Section 2.6), three independently fabricated films per method constitute the fabrication replicates (*n* = 3 films per method), and the 20 punched samples measured per film are technical replicates nested within each film. Intra-film standard deviation is reported per film as a descriptive measure of within-film uniformity, and inter-film standard deviation is computed across the three per-film mean thicknesses (*n* = 3) as a descriptive measure of batch-to-batch variability; (iii) in the single-layer release study (Section 2.10), two independently fabricated films per condition constitute the fabrication replicates and the three punched specimens taken from each film are technical replicates, so aggregate profiles rest on six specimens drawn from two fabrication units and those six specimens are not statistically independent; (iv) in the atomic force microscopy and confocal Raman comparisons (Section 2.7 and 2.8), one film per fabrication method was examined, with three non-overlapping regions imaged per sample by AFM and a single nine-point map acquired per film by Raman, so these comparisons rest on a single fabrication replicate per method; and (v) in the multilayer device release study (Section 2.11), six independently fabricated devices per condition constitute six independent experimental units (*n* = 6 devices).

Three inferential comparisons are reported. First, intra-film thickness standard deviation was compared between fabrication methods by Welch’s unequal-variance t-test, with the independently fabricated film as the experimental unit (*n* = 3 films per method). Second, mean film thickness was compared between methods by the same test (*n* = 3 films per method), in order to test whether the two routes differ in the average dimension achieved as distinct from its variability. Third, the spatial variance of the confocal Raman CAP/F-127 peak-height ratio was compared between the cast and spun maps by an F test on the sample variances of the nine measurement points comprising each map (df = 8, 8). All tests were two-sided and *p* < 0.05 was taken as the threshold for statistical significance. Because the Raman comparison rests on a single mapped region on one film per method, it tests whether compositional heterogeneity differs across the mapped areas of these two particular films; it does not license inference about film-to-film variability in the underlying populations, and it is reported on that restricted basis. Data are presented as mean ± standard deviation with the applicable n stated in each figure legend, and individual film or device traces are shown wherever practical, so that within-group dispersion is visible rather than compressed into a single error bar.

## Results

### Spin-curve characterization and thickness control

**Fig. 2:**
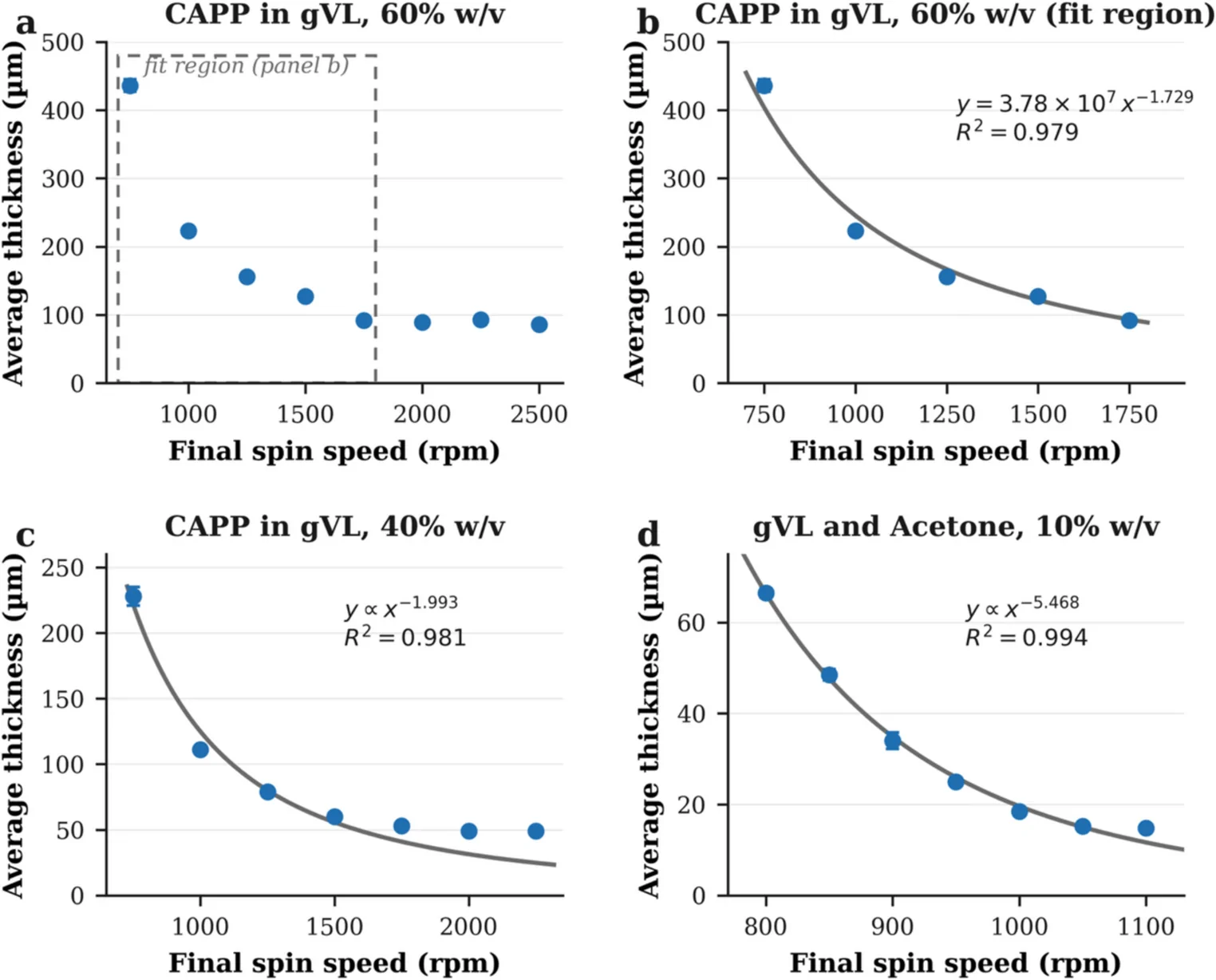
Spin curves for CAPP films deposited on silicon wafers. (**a**) CAPP dissolved in gVL at 60% w/v and spun at varying rpm with constant acceleration; average thickness reported as the mean of five repeated measurements at different locations on a single film (*n* = 5 measurements per film). (**b**) Subset of panel a re-plotted over the varying region to improve power-fit R². (**c**) CAPP dissolved in gVL at 40% w/v. (**d**) CAPP dissolved in a 1:1 gVL/acetone mixture at 10% w/v

To establish the relationship between spin parameters and film thickness for the CAPP system, spin curves were generated by systematically varying rotational speed at a fixed 30 s spin time across multiple solution formulations. For the primary fabrication formulation, CAPP dissolved in gVL at 60% (w/v), film thickness decreased monotonically from 436 ± 9 μm at 750 rpm to 92 ± 4 μm at 1750 rpm (Fig. 2a). The varying region of the curve, spanning approximately 750 to 1750 rpm, was re-plotted with a power-law fit to quantify this relationship (Fig. 2b), confirming that thickness follows an inverse power-law dependence on rotational speed consistent with classical spin coating theory (y = 37,756x⁻¹·⁷²⁹ with thickness in mm, equivalently 3.78 × 10⁷x⁻¹·⁷²⁹ with thickness in µm; R² = 0.979). The fitted exponent of − 1.73 is of the order expected from hydrodynamic treatments of spin coating, in which the balance of centrifugal thinning against viscous drag yields an inverse power-law dependence of final thickness on rotational speed with an exponent near − 0.5 to − 2 depending on whether evaporation is rate-limiting (Meyerhofer 1978; Routh and Russel 1998), and it is consistent with empirical spin curves reported for other free-standing biodegradable polymer films, in which thickness likewise decreased monotonically with rotational speed and increased with solution concentration and viscosity (Allaf 2025). The 10% (w/v) gVL: acetone formulation is a clear exception, fitting an exponent near − 5.5 (Fig. 2d); a dependence this steep is not expected from flow-dominated thinning and is more consistent with an evaporation-limited regime, in which the volatile acetone fraction sets the film thickness before centrifugal thinning is complete. The design space established here is therefore empirical rather than a single physical law spanning all three formulations. The standard deviation across the five within-film measurements also decreased with increasing rotational speed across this range, from 9 μm at 750 rpm to 4 μm at 1750 rpm, a reduction of approximately 56% in absolute terms, indicating that higher rotational speeds produce not only thinner films but films of lower absolute thickness dispersion. Expressed as a coefficient of variation the trend reverses (2.1% at 750 rpm versus 4.3% at 1750 rpm), so the improvement is in absolute rather than relative uniformity. Based on this calibration, 1050 rpm and 1750 rpm were selected as operating conditions for target thicknesses of approximately 400 μm (achieved by two sequential depositions at 1050 rpm) and 100 μm, respectively, for subsequent fabrication and comparison studies.

To evaluate the effect of solution concentration on spin-curve behavior, an additional curve was generated for CAPP dissolved in gVL at 40% (w/v) (Fig. 2c). At equivalent rotational speeds, the lower-concentration formulation produced substantially thinner films, ranging from 228 ± 7 μm at 750 rpm to 49 ± 1 μm at 2000 rpm, demonstrating that solution concentration provides an additional independent parameter for tuning target thickness beyond rotational speed alone. A third formulation consisting of CAPP dissolved in a 1:1 gVL: acetone mixture at 10% (w/v) was also characterized (Fig. 2d), yielding films ranging from 67 ± 1 μm at 800 rpm to 15 ± 1 μm at 1050 rpm, extending the accessible thickness range into the sub-20 μm regime. Together, these spin curves establish that film thickness in the CAPP system can be tuned across more than an order of magnitude by modulating rotational speed and solution concentration in combination, providing a flexible fabrication design space for engineering multilayer constructs with diverse layer thickness requirements.

### Characterization of film uniformity: spin coating vs. cast method

**Fig. 3:**
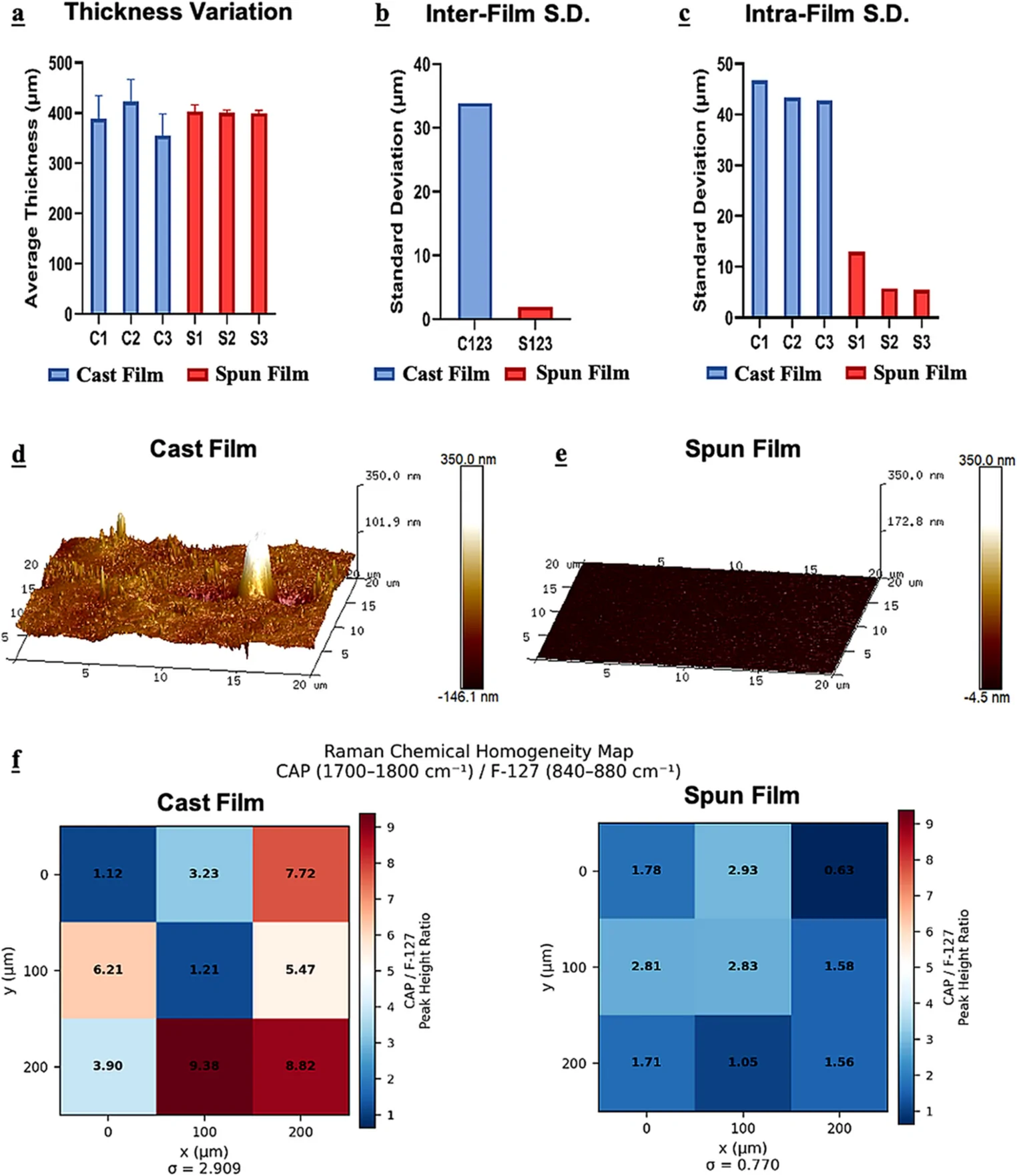
Characterization of film uniformity: solvent casting vs. spin coating. (**a**) average thickness of three cast films (C1, C2, C3) and three spun films (S1, S2, S3), each measured at 20 punched locations; the film is the independent fabrication replicate (*n* = 3 films per method) and the 20 within-film measurements are technical replicates nested within it (Section 2.12). Error bars represent the standard deviation of the 20 within-film measurements. (**b**) inter-film standard deviation of average thickness across the three films per method, reflecting batch-to-batch variability. (**c**) intra-film standard deviation of thickness measurements within each individual film, reflecting within-film uniformity. (**d**) AFM surface topography image of solvent-cast film. (**e**) AFM surface topography image of spun film. (**f**) confocal Raman homogeneity maps of cast and spun films, one film per fabrication method, showing the spatial distribution of the CAP/F-127 peak-height ratio across a 0.3 × 0.3 mm area subdivided into a 3 × 3 grid of equally spaced measurement points

Film thickness uniformity was evaluated across three independently fabricated films per method, with 20 thickness measurements collected from each film (Fig. 3). Average film thicknesses across all six films were comparable at the 400 μm target, indicating that both solvent casting and spin coating can achieve the intended nominal dimension (Fig. 3a), though cast films displayed substantially larger standard deviation than spun films, indicating greater within-film thickness variation even at matched average values.

Inter-film standard deviation, reflecting variability in average thickness across independently fabricated batches, was approximately 34 μm for cast films and approximately 2 μm for spun films (Fig. 3b), representing an approximately 17-fold reduction in batch-to-batch variability with spin coating. Because this quantity is a single dispersion value per method, computed across the three per-film mean thicknesses, it is a ratio of two point estimates and is reported descriptively; it is not amenable to statistical testing at this level of replication (Section 2.12). Mean thickness itself did not differ detectably between the two methods (Welch’s unequal-variance t-test on the three per-film mean thicknesses, *p* = 0.63). At *n* = 3 films per method this test is underpowered and the null result is not evidence of equivalence, but it does indicate that the difference between the routes is not a gross offset in the average dimension achieved. Intra-film standard deviation, reflecting thickness variation within individual films, ranged from approximately 43 to 47 μm across cast films C1, C2, and C3, while spun films S1, S2, and S3 exhibited intra-film standard deviations of approximately 12, 6, and 5 μm respectively (Fig. 3c), representing an approximately three- to nine-fold reduction in intra-film variability for spun films. Treating the film as the experimental unit, the reduction in intra-film standard deviation was significant (Welch’s unequal-variance t-test on the three per-film standard deviations per method, *p* < 0.01).

Surface topography was further evaluated by atomic force microscopy (AFM) to assess nanoscale morphological differences between fabrication methods (Fig. 3d and e). The solvent-cast film surface exhibited a highly irregular topography across the 20 × 20 μm scan area, with numerous sharp protrusions distributed across the field. Both images are displayed with the same upper limit of 350.0 nm; the lower limit of each color scale differs between the two and is the deepest excursion below the mean plane recorded in that field. For the cast surface that limit is 146.1 nm (Fig. 3d). In contrast, the spun film surface, imaged over the same 20 × 20 μm scan area and displayed with the same upper limit, appeared essentially planar with only faint directional striations attributable to spin direction and a corresponding limit of only 4.5 nm below the mean plane (Fig. 3e), a factor of approximately 32 smaller than the cast film on the same measure. Because AFM was performed on one film per fabrication method, with three non-overlapping regions imaged per sample, this comparison rests on a single fabrication replicate per method and is presented as a qualitative difference in surface morphology rather than a quantified population-level effect (Section 2.12). The near-flat topography of the spun film is consistent with the centrifugal spreading mechanism enforcing uniform solvent evaporation and polymer deposition across the substrate (Meyerhofer 1978; Routh and Russel 1998), although the two routes also differ in solvent, concentration and drying conditions (Section 2.4), so the deposition step cannot be isolated as the sole cause.

Raman mapping was performed on both cast and spun films across a 0.3 × 0.3 mm area subdivided into a 3 × 3 grid of nine equally spaced measurement points (Fig. 3f). Spectral integration windows were selected to capture chemically distinct signatures of each blend component: the CAP carbonyl stretch (1700 to 1800 cm⁻¹) and the F-127 C-O-C stretch (840 to 880 cm⁻¹), both of which are well-resolved from one another and from background contributions in the CAPP spectrum. At each measurement point, a CAP/F-127 peak-height ratio was calculated by dividing the maximum intensity above a local linear baseline within the CAP window by the equivalent baseline-corrected peak height within the F-127 window, producing a spatially resolved index of relative component abundance across the film surface.

The cast film exhibited CAP/F-127 ratios ranging from 1.12 to 9.38 across the 9 measurement points, with a standard deviation of 2.909, reflecting substantial point-to-point variability in local polymer composition. The spun film, by contrast, produced ratios ranging from 0.63 to 2.93, with a standard deviation of 0.770, approximately 3.8-fold lower than that of the cast film. The spatial variance of the peak-height ratio was significantly lower across the spun map than across the cast map (F(8,8) = 14.2, *p* = 0.0011). Because each map represents a single 0.3 × 0.3 mm region on one film per method, this test establishes that compositional heterogeneity differs across the mapped areas of these two particular films and does not by itself support inference about film-to-film variability (Section 2.12). The spatial distribution of these ratios, visualized on a shared color scale in Fig. 3f, shows that the cast film contains regions of pronounced CAP enrichment and F-127 depletion within a single 0.3 × 0.3 mm area, a pattern consistent with phase separation or incomplete mixing during solvent evaporation casting. Composition-dependent component distribution has previously been reported for CAP-based composite films and shown to depend on the evaporation regime (Jeon et al. 2007; Sundararaj et al. 2013; Xu and Lee 1993), and greater non-uniformity in gravity-leveled cast films than in centrifugally spread films is consistent with published spin-coating characterizations of biodegradable polymer systems (Allaf 2025). Nonetheless, surface Raman mapping of a single region cannot distinguish between these possibilities or exclude a localized surface artifact. The spun film, while not perfectly uniform, displays substantially more consistent ratios across the mapped area, consistent with the homogenizing effect of centrifugal shear during deposition (Meyerhofer 1978; Routh and Russel 1998), subject to the same caveat that the two routes differ in formulation as well as in deposition mechanism (Section 2.4).

### Single-layer drug release kinetics

**Fig. 4:**
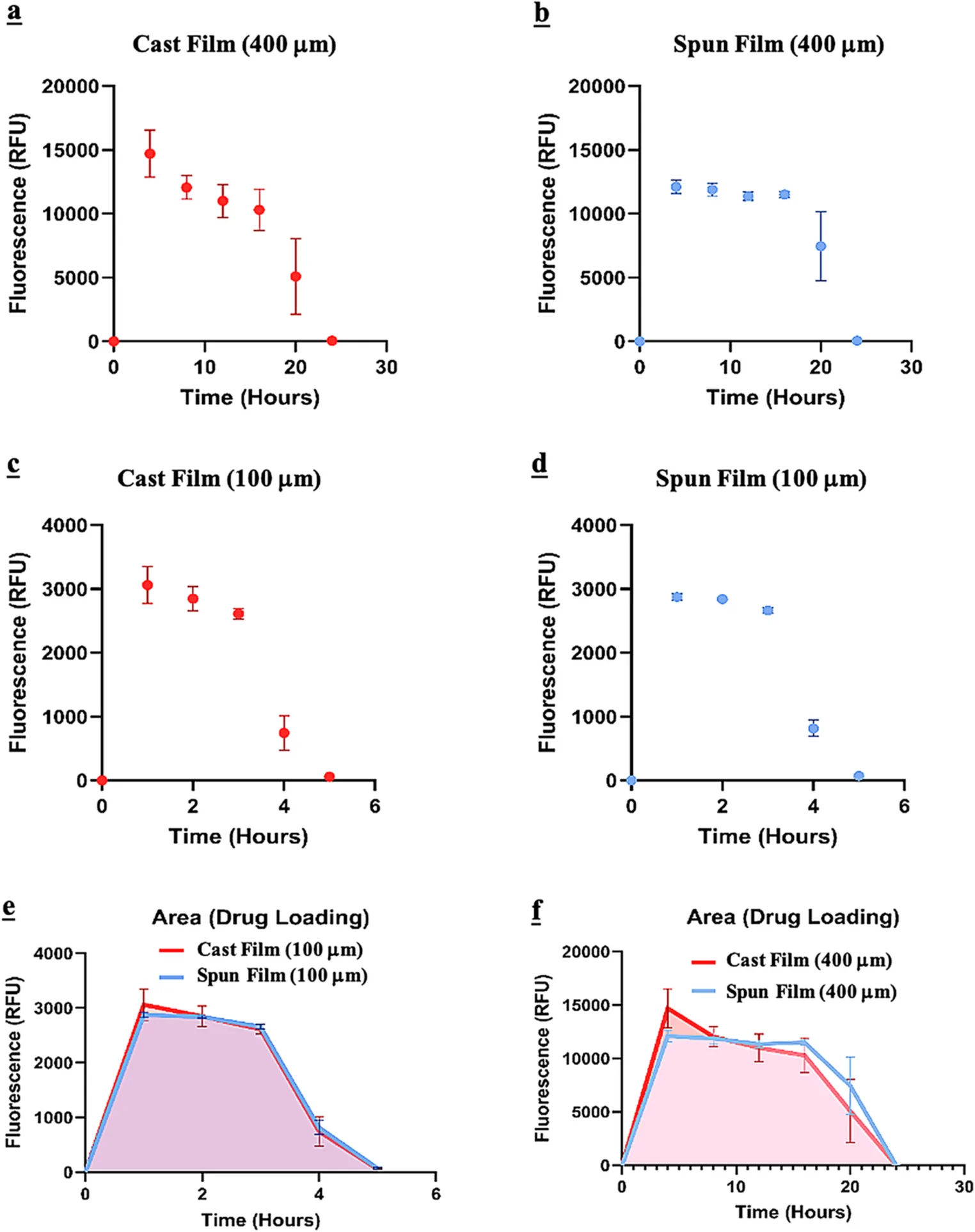
In vitro release profiles and comparative drug loading: solvent casting vs. spin coating at two film thicknesses.(**a**,**b**) fluorescence intensity (RFU) over time for cast and spun films fabricated at a target thickness of 400 μm, respectively; error bars represent standard deviation. For all release panels, *n* = 6 specimens per condition drawn from two independently fabricated films; the three specimens from a given film are technical replicates nested within that film (Section 2.12). (**c**, **d**) fluorescence intensity over time for cast and spun films at a target thickness of 100 μm, respectively; error bars represent standard deviation. (**e**) overlay of release profiles for cast and spun films at 100 μm, with shaded area under the curve used as a proxy for total drug (fluorescent dye) loading per film. (**f**) overlay of release profiles for cast and spun films at 400 μm

Single-layer rhodamine-B-loaded films at both 400 μm and 100 μm target thicknesses were evaluated for in vitro fluorescent dye release in PBS (pH 7.4) at 37 °C to compare release behavior between fabrication methods (Fig. 4). For 400 μm films, cast films released most of their payload early, reaching approximately 14,800 RFU at the first 4 h sampling point and declining monotonically thereafter (Fig. 4a). Spun films at the same thickness displayed a markedly different kinetic profile, holding a plateau of approximately 11,300 to 12,100 RFU from 4 to 16 h, falling to approximately 7,500 RFU at 20 h, and reaching baseline by 24 h (Fig. 4b). Both methods achieved complete release by 24 h. Variability was substantially lower in spun films, with average inter-sample standard deviation approximately half that of cast films (622 RFU vs. 1,242 RFU).

For 100 μm films, complete release occurred within 5 h for both methods (Fig. 4c, d). Both profiles declined gradually from a first-hour maximum near 3,000 RFU through 3 h before falling to baseline by 5 h; at this thickness the two methods differ in dispersion rather than in the shape of the release curve. Within-group standard deviation for spun 100 μm films was approximately 31% of that observed for cast films across the sampled release period.

The near-constant release rate of the 400 μm spun films invites mechanistic interpretation, although the mechanism is not established by these data. The profile is consistent with uniform dye distribution throughout the film matrix, where release proceeds at a near-constant rate until near-complete depletion triggers rapid signal loss. In contrast, the early-weighted profile of the 400 μm cast films is consistent with heterogeneous drug distribution, where surface-concentrated or loosely incorporated drug releases rapidly at early timepoints, followed by slower diffusion from less accessible regions of a structurally irregular matrix. A transition of this kind between diffusion-dominated early release and erosion-controlled later release has been described for CAP–Pluronic systems, in which the dominant mechanism depends on the local hydration environment and on how the payload is distributed within the matrix (Arora et al. 2006; Xu and Lee 1993). The release measurements report only bulk fluorescence in the supernatant and carry no depth-resolved information about dye distribution, so a surface-concentrated dye population is inferred from the shape of the release curve rather than observed. The AFM and thickness data in Fig. 3 establish that cast films are more structurally heterogeneous, which is consistent with a corresponding heterogeneity in dye distribution. Other contributors include differences in film density and free volume arising from the different drying regimes, a larger effective surface-area-to-volume ratio in a rougher film, residual solvent, and differences in the initial extent of polymer–dye phase separation. Depth-resolved compositional mapping of film cross-sections would be required to test the surface-concentration hypothesis directly; these mechanisms are therefore offered as hypotheses rather than conclusions.

Overlay of release profiles with shaded area under the curve indicated comparable total dye loading between methods at both thicknesses, consistent with the interpretation that the observed differences in release shape reflect fabrication-driven structural differences rather than differences in loading (Fig. 4e, f). The convergence of AUC values across methods argues against total loading as the explanation for the difference in profile shape at 400 μm and thereby points to film architecture as the more likely determinant of release kinetics, with spin coating producing more reproducible release behavior at both thickness targets evaluated. Because AUC here is a proxy computed from fluorescence in the collected supernatant rather than a direct mass balance, it constrains rather than proves equivalence of loading.

### Multilayer device architecture and interfacial morphology

**Fig. 5:**
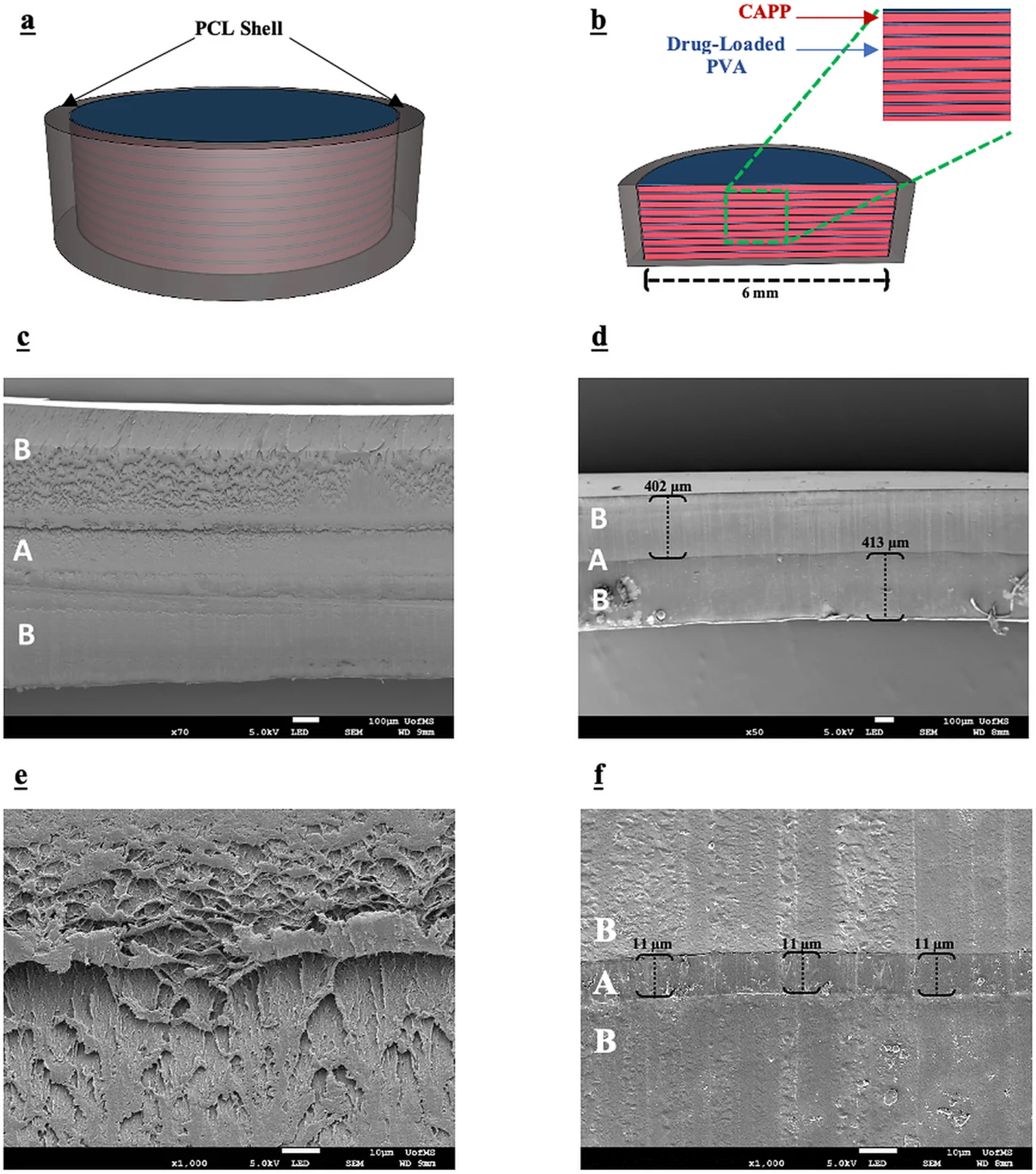
CAD renderings and scanning electron microscopy (SEM) images of the multilayer CAPP implant construct and three-layer test samples fabricated by solvent casting and spin coating.(**a**) exterior CAD rendering of the full implant device showing the PCL sheath and cylindrical geometry. (**b**) cross-sectional CAD rendering of the internal alternating blank (CAPP) and active (fluorescein-PVA) layer architecture. (**c**) cross-sectional SEM image of a solvent-cast three-layer construct (nominal 400–100–400 μm; blank CAPP–rhodamine-B–loaded CAPP–blank CAPP). (**d**) cross-sectional SEM image of a spin-coated blank–active–blank construct (nominal 400–10–400 μm). In the spin-coated cross-sections (d), labels A and B denote the fluorescein-PVA active layer and the blank CAPP layers, respectively; in the cast cross-sections (c), the middle layer is a rhodamine-B–loaded CAPP layer. (**e**) higher-magnification view of the solvent-cast middle-layer interface. (**f**) higher-magnification view of the spin-coated active-layer interface. The A and B annotations appear on both the cast and the spin-coated panels but denote different materials and carry different evidential weight. In (d) and (f), A is the fluorescein-PVA active layer and B the blank CAPP layers, and the labels mark interfaces directly resolvable in the image; the accompanying thickness annotations are measurements made from those images. In (c), A is the rhodamine-B-loaded CAPP middle layer and B the blank CAPP layers, but there the labels indicate the approximate positions of the nominal layers inferred from the deposition and bonding sequence, because the contrast boundaries in the cast construct could not be unambiguously assigned to layer interfaces. No A or B labels appear in (e): at ×1,000 the cast cross-section presents continuous interfacial disruption with no boundary assignable to a specific layer, so no region can be identified as active or blank

The full implant architecture consists of alternating CAPP blank layers and thin fluorescein-PVA active layers encapsulated within a PCL sheath. The exterior device geometry and internal layered composition are illustrated in Fig. 5a and b as CAD renderings, providing design-level context for the cross-sectional characterization that follows.

SEM was used to evaluate interfacial definition and layer morphology in three-layer CAPP constructs fabricated by solvent casting and spin coating (Fig. 5c–f). In the spin-coated cross-sections, labels A and B denote the fluorescein-PVA active layer and the blank CAPP layers, respectively; in the cast cross-sections, the middle layer is a rhodamine-B–loaded CAPP layer. These two constructs are not compositionally or dimensionally matched. The cast construct contains a 100 μm rhodamine-B-loaded CAPP middle layer because 100 μm approaches the practical lower thickness limit of solvent casting for this system, whereas the spin-coated construct contains a 10 μm fluorescein-loaded PVA middle layer of the composition actually used in the functional devices. The two constructs also differ in assembly route, the cast one having been joined by post hoc acetone bonding of three separately cast films and the spin-coated one built by sequential in situ deposition. Figure 5c–f therefore show the interfacial quality each route achieves at its own practical limit rather than a one-to-one structural comparison, and the differences observed cannot be attributed to the deposition mechanism alone. Both constructs additionally contain fewer layers than the Q16 and Q72 devices characterized functionally in Section 3.5, so the interfacial quality shown here should not be assumed fully representative of the complete multi-pulse stacks.

Solvent-cast three-layer samples (400–100–400 μm) (Fig. 5c) were fabricated at the practical lower limit of solvent casting for CAPP films; unlike spin coating where thinner films exhibit reduced thickness variability, solvent casting becomes increasingly unreliable below approximately 100 μm due to amplified sensitivity to evaporation gradients, surface tension effects, and humidity, and the mechanical challenge of removing an intact thin film from a petri dish without tearing or deformation introduces additional sources of error that compound at reduced thickness. This constraint represents a practical limitation of solvent casting relative to spin coating. These cast samples exhibited substantial variation in interfacial structure and layer definition. Layer boundaries could not be unambiguously resolved from cross-sectional imaging alone; the apparent contrast boundaries did not correspond to the expected thickness ratio, and the upper blank layer displayed visibly rougher surface morphology than the lower blank layer, suggesting asymmetric interfacial disruption during acetone-mediated bonding. The middle CAPP layer exhibited an apparent average thickness of 225 μm, substantially exceeding the intended 100 μm, with high spatial variability across measurement locations. Because the boundaries themselves could not be unambiguously assigned, this value is the extent of the region of altered contrast rather than a resolved layer thickness. Higher magnification imaging (Fig. 5e) revealed extensive interfacial disruption, including surface folding, laminar separation, and zones of apparent delamination. The disruption at this magnification is continuous across the field, and no boundary could be assigned to a specific layer; Fig. 5e therefore carries no region labels. The observation carries structural information: in a construct intended to consist of three discrete layers, the inability to identify any layer boundary at 10 μm scale indicates that acetone-mediated bonding did not produce interfaces resolvable at this scale, although cryosectioning artifacts cannot be excluded as a contributor in the absence of a sectioning control. The labels applied to the lower-magnification cast panel (Fig. 5c) should correspondingly be read as indicating where the nominal layers are expected from the assembly sequence, not as boundaries resolved in the image. Because this construct was assembled by post hoc solvent bonding whereas the spin-coated construct was built by sequential in situ deposition, the interfacial disruption seen here is attributable to the assembly route at least as much as to the film-formation method itself.

Spin-coated three-layer samples (400–10–400 μm) (Fig. 5d) exhibited well-defined and continuous interfaces between the CAPP blank layers and the fluorescein-PVA active layer. The active layer appeared uniform across the interface, with an average measured thickness of 11 μm across three spatially separated measurement locations, closely matching the intended 10 μm deposition thickness. The two blank CAPP layers flanking it are annotated on the same panel at 402 μm and 413 μm against a 400 μm nominal target (Fig. 5d), corresponding to deviations of 0.5% and 3.3% respectively, and are consistent with the thickness fidelity inferred from the dial-indicator measurements in Section 3.2. Because a 400 μm blank unit is deposited as two sequential 200 μm layers, this construct reproduces both interface types found in the complete devices, and the continuity resolved here is the continuity on which the multilayer stacks depend. As these are measurements on a single construct, they corroborate rather than independently establish that result. Higher magnification imaging (Fig. 5f) showed smooth and continuous transitions between layers, with no delamination, surface disruption, or boundary irregularity visible at the magnifications and in the regions imaged; absence of such features in the imaged fields does not establish their absence throughout the construct. The consistency of both layer thickness and interfacial morphology across measurement locations supports the reproducibility of spin coating as a fabrication strategy for thin, discrete active layers within multilayer constructs.

### Pulsatile release at two inter-pulse intervals

**Fig. 6:**
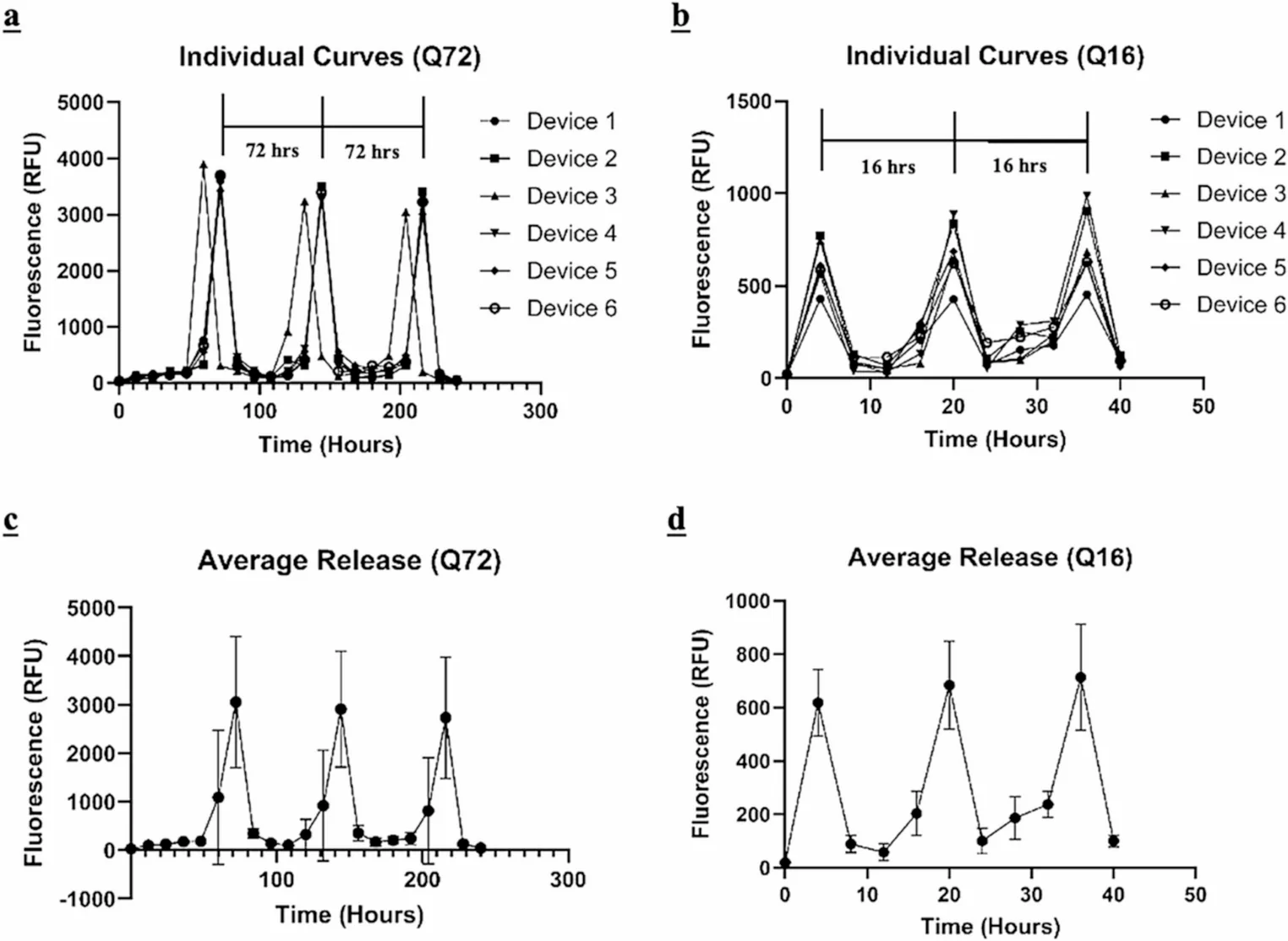
Pulsatile in vitro release profiles at two inter-pulse intervals.(**a**) Individual fluorescence release curves for six Q72 devices, with bracketed annotations indicating the measured 72 h inter-pulse periods, which matched the design closely. (**b**) individual fluorescence release curves for six Q16 devices, with bracketed annotations indicating the measured approximately 16 h inter-pulse spacing (Section 3.5). (**c**) average release profile across all six Q72 devices; error bars represent standard deviation. Elevated standard deviation at select timepoints reflects a single-device phase offset in pulse initiation rather than variability in peak amplitude or dye release magnitude. (**d**) average release profile across all six Q16 devices; error bars represent standard deviation

To evaluate whether blank layer thickness governs inter-pulse timing as designed, multilayer devices were fabricated and tested in two configurations, designated Q72 and Q16 after the inter-pulse intervals they produced. Individual release curves for six devices per condition are shown in Fig. 6a and b, respectively. The brackets in Fig. 6a and b mark the measured inter-pulse periods in each case.

Q72 devices exhibited three discrete, well-separated fluorescence peaks, recorded at 72, 144, and 216 h, giving successive inter-pulse intervals of approximately 72 h and so matching the design target (Fig. 6a). The position of the first peak is set by the blank unit that forms the exposed face of a Q72 device, so the interval from immersion to first release is itself a design-defined quantity in this condition. Peak fluorescence values ranged from approximately 3,000 to 4,500 RFU across devices and pulses, with inter-pulse fluorescence returning to near-zero baseline between each release event, indicating effective suppression of dye diffusion during blank layer degradation intervals. Q16 devices similarly produced three clearly defined, well-separated release events at 4, 20, and 36 h, with peak fluorescence ranging from approximately 500 to 1,000 RFU and comparable inter-pulse baseline suppression (Fig. 6b). The observed inter-pulse spacing was therefore approximately 16 h. The 400 μm barrier used in these devices was designed to give a 12 h inter-pulse interval, so the interval measured exceeded the design intent by approximately 33%. Q16 devices thus generated reproducible, discretely separated pulses, at an interval slightly longer than the barrier geometry was intended to produce.

Average release profiles across all six devices per condition are shown in Fig. 6c and d for Q72 and Q16, respectively. The elevated standard deviation visible in the Q72 average profile (Fig. 6c) does not reflect high variability in pulse amplitude or drug release magnitude across devices. Inspection of the individual curves (Fig. 6a) reveals that peak shapes and amplitudes are highly consistent across the six devices; the inflated error at certain timepoints is attributable to a single device initiating its release cycle one measurement interval earlier than the remaining five, producing a phase offset that broadens the averaged peak and artificially elevates the standard deviation at that timepoint. A single device beginning one sampling interval earlier is consistent with device-to-device variation in the thickness of the outer blank unit, which shifts the whole pulse train without altering the intervals within it. Averaging across devices therefore broadens peaks even when inter-pulse timing is reproducible. When interpreted in the context of individual curves, inter-device timing reproducibility and pulse amplitude consistency are both well preserved across the Q72 cohort. Inter-device variability in peak timing was similarly low for Q16 devices, with pulse timing appearing more reproducible across devices than pulse amplitude, the latter reflecting expected device-to-device variation in active layer drug loading rather than structural timing inconsistency.

Two features of these measurements bear on the interpretation of the timing behavior. First, the interval between immersion and the first release event is set by the layer that forms the exposed face, and the two conditions differ in that layer (Section 2.5). A Q72 device carries a full 2.4 mm blank unit at the exposed face, so the first release is expected only after that unit has eroded, and it was recorded at 72 h. A Q16 device presents an active layer at the exposed face, so release begins on immersion, and the first peak was recorded at the first 4 h sampling point. The interval to first release is therefore fabrication-defined in both conditions and is interpreted alongside the intervals between successive pulses. Second, the temporal sampling resolution differs substantially between the two conditions relative to the interval each was designed to produce: supernatant was collected every 4 h for Q16 devices, one third of the intended 12 h design target, but every 12 h for Q72 devices, one sixth of the 72 h target. Both inter-pulse values therefore carry a quantization uncertainty of one sampling interval, so the agreement in the Q72 case is established only to within that resolution.

With those qualifications, the quantity to be explained is that a nominal 400 μm barrier gave inter-pulse intervals of approximately 16 h against a 12 h target, while a nominal 2.4 mm barrier gave intervals consistent with its 72 h target. One class of explanation can be excluded at the outset. A proportional error, whether in the thickness delivered per deposition or in the erosion rate assumed for the calculation, would lengthen both conditions by the same fraction and so cannot produce a 33% overshoot at 12 h alongside agreement at 72 h. The deviation is not proportional to barrier thickness, and any adequate explanation must account for that.

The explanation we favor is a difference in solvent removal between the two device conditions, whose effect is not proportional to barrier thickness. As described in Section 2.5, the Q72 devices were vacuum dried at room temperature for 24 h before testing, whereas the earlier Q16 devices received no drying beyond the overnight step that follows encapsulation. Residual solvent retained in an incompletely dried device is a blend of indeterminate composition, predominantly gVL from the barrier depositions together with chloroform and acetone able to penetrate the bulk during PCL spray coating, and its removal from the bulk is slow, depending on humidity and solvent vapor pressure rather than on elapsed time, so an overnight step alone is unlikely to complete it. In our experience with this system, devices carrying a residual solvent burden have eroded more slowly in phosphate-buffered saline than fully dried devices of comparable geometry, and a solvent-plasticized matrix at the eroding face would be expected to present different water activity and chain mobility than a fully dried one. We advance this as the most plausible account of the Q16 offset rather than as a demonstrated mechanism: residual solvent was not quantified in these devices, and no measurement reported here isolates its contribution. It does, however, predict both the direction of the discrepancy and its non-proportional dependence on barrier thickness, since a given retained solvent burden occupies a larger fraction of a 400 μm barrier’s erosion time than of a 2.4 mm barrier’s, which a proportional error cannot.

The dataset contains a comparison bearing on this account. Reduced-pressure drying was adopted as the standard final step and the Q72 devices were prepared under it; their agreement with the 72 h design target, within the one-interval sampling resolution noted above, is consistent with timing accuracy being obtained once solvent removal is complete. At the shorter interval, a 400 μm barrier yielded three discrete, well-separated release events with near-baseline inter-pulse suppression and low inter-device variability, at an absolute interval approximately 33% longer than the design target. Completeness of solvent removal is therefore a process variable that must be specified alongside layer thickness when a target interval is translated into a fabrication recipe.

Collectively, these results show that inter-pulse timing increased with blank layer thickness across the two design targets tested, consistent with the surface-erosion-controlled release of CAP–Pluronic composites at physiological pH on which the design of this architecture rests (Hossain et al. 2023; Xu and Lee 1993), and that near-complete inter-pulse suppression is achievable at both intervals tested. Timing matched the design target for the 72 h condition, within the sampling resolution, and was reproducible but approximately 33% longer than the design intent for the Q16 condition, indicating that although the ordering and reproducibility of pulses are governed reliably by fabrication geometry, the accuracy of the absolute interval also depends on process variables identified above.

## Discussion

Across every metric examined, spin coating produced more uniform films than solvent evaporation casting. Spin coated films varied less in thickness, chemical and topological heterogeneity, and drug release profiles compared to solvent casted films. These differences are consistent with the distinct film-formation dynamics of the two routes, albeit the two routes also differ in solvent, concentration and drying conditions (Section 2.4): solvent evaporation during casting introduces concentration gradients and surface instabilities as the drying front propagates, whereas centrifugal thinning promotes uniform radial flow and simultaneous solvent removal (Meyerhofer 1978; Routh and Russel 1998). In multilayer constructs, sequential deposition produced continuous, well-defined interfaces with no interlayer mixing detectable at the magnifications imaged, whereas cast constructs joined by acetone bonding showed interfacial disruption and extremely heterogenous barriers (Fig. 5). Consistency of interval timing and homogeneity of pulse AUCs was radically improved with the spin coating technique introduced here as compared to our previous attempts at assembling multilayered solvent casted films. Though we initially designed the Q16 devices to have 12 h intervals, the resulting films exhibited 16 h intervals instead. It is likely that the interval was missed by ~ 33% in these films because of the residual solvent retained in the films from the successive spin coating process, which in these devices were tested before the extended vacuum drying step was adopted. It is likely that this contributed more to the Q16 devices because of the thinner interval layers, but further studies are needed to fully probe the impact of solvent retention on multilayer film degradation.

The performance characteristics of multilayered spin coated films compare favorably to pulsatile release profiles generated from solvent-cast CAPP multilayer devices reported in a recent publication from our group, which demonstrated signal attenuation across successive doses and elevated inter-pulse background attributable to drug diffusion through cast interfaces (Hossain et al. 2023). The spin-coated constructs described here produced improved signal-to-background ratios, pulse breadth, and pulse consistency, suggesting that the interfacial precision conferred by spin coating may translate into improved release fidelity relative to cast architectures. This comparison is drawn between separate studies performed with different payloads, device geometries, and sampling schedules rather than under matched conditions, and therefore necessitates confirmation in future studies.

Set against the wider pulsatile literature, this architecture occupies a distinct position on the trade-off between programmability and hardware. Microfabricated reservoir implants achieve programmable multi-pulse dosing and, in their wirelessly controlled form, retain control over dose timing after implantation (Farra et al. 2012; Prescott et al. 2006; Richards Grayson et al. 2003; Santini et al. 1999). That capability is obtained at the cost of a sealed compartment and a membrane per dose, with onboard electronics in the actuated versions, so the achievable pulse count scales with device volume and release depends on hardware continuing to function in vivo. In the architecture developed here, pulse count is set by the number of deposited active layers and release is governed merely by biodegradation of the device components. Therefore, no power source or actuator is required, and no explant surgeries would be needed for the fully biodegradable constructs. A trade-off of the current devices, like all passive, biodegradable systems, is that the release schedule is fixed upon fabrication and cannot be revised in vivo. Oral rupture and compression-coated systems avoid hardware in the same way but are generally limited to one or two bursts, and their lag times remain sensitive to gastrointestinal pH, transit and hydration (Arora et al. 2006; Bussemer et al. 2001; Hata et al. 1994), whereas the interval here is set by a fabricated dimension in a controlled implant environment.

By enabling deterministic control over layer thickness, spin coating enables temporal programming according to fabrication-defined geometry. Furthermore, the use of pharmaceutically established polymers such as CAP and Pluronic F-127 supports translational feasibility. However, because every barrier deposition carries a 2 h bake at 90 °C, thermal history accumulates with the number of depositions a device requires. Therefore, the current manufacturing process developed here is only suitable for pharmaceutical agents that can withstand significant heating. Two routes to reducing or eliminating that heat dependency are worth exploring: vacuum drying at room temperature in place of oven / hot-plate baking, and flash-spinning thinner layers whose solvent evaporates essentially instantaneously without heating.

The present study established programmable release across three pulses at two clinically relevant intervals; extending the same approach to a greater number of active layers is a natural next direction. Reaching weekly or monthly dosing windows would, however, require a higher volumetric drug density or a slower-eroding barrier chemistry rather than simply more barrier thickness at the present erosion rate. Functional validation of such extended-schedule devices, with visualization of their internal architecture, will be needed to define the practical upper bound on pulse number and to confirm that timing fidelity is preserved across deeper stacks. Substituting a therapeutic molecule for the fluorescent tracer used here and sizing the encapsulating shell that sets the outer dimensions of the construct, are the remaining steps toward an implantable form. Two developments would extend the approach further. Machine learning methods now being applied across drug discovery and formulation offer a route to predicting erosion rate and interval from composition and deposition parameters, which would replace the empirical calibration used here with a model-guided design step (Basak 2026a, b). Combining a geometrically encoded schedule with stimulus-responsive barrier chemistries would additionally allow a pre-programmed sequence to be modulated in response to the local environment rather than fixed entirely at fabrication; bioinspired phenolic polymer networks developed for locally acting antioxidant and anti-inflammatory therapy are one source of candidate chemistries (Basak and Das 2026).

Importantly, the principles demonstrated here may extend beyond CAPP to other surface-eroding polymers. Early work on polyanhydrides, including poly(sebacic anhydride) (PSA), established that these materials degrade via surface erosion, producing relatively constant mass loss under ideal geometric conditions (Shieh et al. 1994; Tamada and Langer 1993). However, prior studies have also demonstrated that apparent degradation rates change as device geometry evolves and exposed surface area decreases over time; in conventional bulk geometries, progressive reduction in exposed surface area can lead to non-linear erosion kinetics and altered pulse timing (Akbari et al. 1998). By contrast, the multilayer architecture described here is designed to maintain a constant exposed surface area during erosion, which should minimize geometry-driven rate changes. A nominally constant exposed area does not by itself guarantee a constant erosion rate: the intervals measured here are not proportional to barrier thickness (Section 3.5), so factors other than exposed area contribute to release timing. Extension of this fabrication strategy to poly(sebacic anhydride) and related surface-eroding polymers may enable reduced device form factors, higher drug density per unit volume, and increased pulse capacity while preserving temporal precision.

## Conclusion

Spin coating provides a reproducible and tunable method for fabricating polymer-based drug delivery films, addressing key limitations of conventional solvent evaporation casting including thickness heterogeneity, interfacial irregularity, and batch-to-batch variability. At a matched 400 μm target it reduced batch-to-batch thickness standard deviation from approximately 34 to 2 μm; at a 100 μm target it reduced the maximum excursion below the mean plane, measured by atomic force microscopy, from 146.1 to 4.5 nm. Confocal Raman mapping showed the standard deviation of the CAP/F-127 peak-height ratio falling from 2.91 to 0.77, and in multilayer constructs it produced continuous interfaces with an active layer measuring 11 μm against a 10 μm target, whereas separately cast and solvent-bonded constructs gave a middle region averaging 225 μm against a 100 μm target. Devices designated Q72 and Q16 each produced three discrete, well-separated release events with near-baseline inter-pulse signal (*n* = 6 devices per condition). Q72 devices gave successive inter-pulse intervals of approximately 72 h, matching their design target within the sampling resolution, whereas Q16 devices gave intervals of approximately 16 h against a 12 h design intent, an overshoot of approximately 33%. Two limitations bound these conclusions. The cast and spin-coated films were necessarily produced from different formulations and drying conditions, since neither route yields usable films under the other’s conditions, so the comparison is between two complete fabrication routes rather than between deposition mechanisms alone. And timing is harder to control at the thinner barriers required for short intervals, where a given error in layer thickness or in solvent removal is a larger fraction of the target interval. As healthcare systems increasingly prioritize adherence and personalization, fabrication platforms that encode dosing schedules geometrically offer an attractive route to consistent dosing with minimal user dependency. In vivo validation is the next step from the present demonstration toward clinically viable, patient-independent pulsatile delivery implants.

## Data Availability

All data supporting the findings of this study are available upon reasonable request from the corresponding author.
